# Financing Maternity and Early Childhood Healthcare in The Australian Healthcare System: Costs to Funders in Private and Public Hospitals Over the First 1000 Days

**DOI:** 10.34172/ijhpm.2020.68

**Published:** 2020-05-12

**Authors:** Emily Callander, Antonia Shand, David Ellwood, Haylee Fox, Natasha Nassar

**Affiliations:** ^1^Menzies Health Institute Queensland, Griffith University, Southport, QLD, Australia.; ^2^Children’s Hospital at Westmead Clinical School, Faculty of Medicine and Health, University of Sydney, Sydney, NSW, Australia.; ^3^Department of Maternal Fetal Medicine, Royal Hospital for Women, Randwick, NSW, Australia.; ^4^School of Medicine, Griffith University, Southport, QLD, Australia.; ^5^School of Nursing and Midwifery, Griffith University, Meadowbrook, QLD, Australia.

**Keywords:** Perinatal Health, Maternal Health, Early Childhood Health, Universal Healthcare, Caesarean Section, Healthcare Costs

## Abstract

**Background:** Maternity care is a significant contributor to overall healthcare expenditure, and private care is seen as a mechanism to reduce the cost to public funders. However, public funders may still contribute to part of the cost of private care. The paper aims to quantify (1) the cost to different funders of maternal and early childhood healthcare over the first 1000 days for both women giving birth in private and public hospitals; (2) any variation in cost to different funders by birth type; and (3) the cost of excess caesarean sections in public and private hospitals in Australia.

**Methods:** This study utilised a whole of population linked administrative dataset, and classified costs by the funding source. The mean cost to different funders for private hospital births, and public hospital births in the Australian state, Queensland are presented by time period and by birth type. The World Health Organization’s (WHO’s) C-model was used to identify the optimal caesarean section rate based upon demographic and clinical factors, and counterfactual analysis was utilised to identify the cost to different funders if caesarean section had been utilised at this rate across Australia.

**Results:** We found that for women who gave birth in a public hospital as a public patient, the mean cost was $22474. For women who gave birth in a private hospital the mean cost was $24731, and the largest contributor was private health insurers ($11550), followed by Medicare ($7261) and individuals ($3312). Private hospital births cost government funders $10050 on average; whereas public hospital *public patient* births cost government funders $21723 on average and public hospital *private patient* births cost government funders $20899 on average. If caesarean section deliveries were reduced, public hospital funders could save $974 million and private health insurers could save $216 million.

**Conclusion:** Private hospital births cost government funders less than public hospital births, but government funders still pay for around 40% of the cost of private hospital births. Caesarean sections, which are more frequently performed in private hospitals, are costly to all funders and reducing them could impart significant cost savings to all funders.

## Background

Maternity care is a unique area of the health system – experiencing high volumes of ‘patients’ and yet not being amenable to prevention or curative initiatives that would see the number of ‘patients’ decline. Due to this large volume, a considerable amount of healthcare resources are consumed by maternity services and this is expected to continue.^1-4^ Multiple countries are actively seeking ways of improving efficiency and productivity in maternity care, either through the provision of interventions and reform at the point of care, or through structural means such as through the way maternity services are funded.^5,6^ The latter is in recognition of the impact that financing mechanisms can have on the costs of care.

As a part of the funding and delivery of healthcare in general, many countries have a two-tier healthcare system. Under such arrangements, women may access care in public hospitals without any out of pocket fees, or at a heavily subsidised rate. Care may alternatively be accessed through private hospitals and paid for or subsidised by private health insurance coverage held by women. Private hospitals and private providers are used to provide women with greater options in the type of care they receive during pregnancy.^7^ Governments may still contribute to part of the costs of private care, but from a public funder perspective, it is generally considered that having private care as a component in a health system reduces the cost to the public funders.^8^ Such arrangements exist in countries such as Australia, New Zealand, Ireland, Italy, Greece, Spain, France, Germany, the Netherlands, and the United Kingdom.^9^

However, with the provision of maternity care, concern has been raised over the intervention rates in private hospitals,^10^ which are considerably higher than in public hospitals in Australia, France, Greece, Italy, and Ireland.^11-15^ Obstetric interventions include caesarean section, instrumental vaginal delivery, induction of labour and episiotomy, with studies showing caesarean section is one of the most common forms of intervention and is used disproportionately among women accessing private care.^11-13^The higher rates of intervention in private hospitals are of concern due to the potential negative long-term health outcomes for mother and child, and high costs, part of which may be incurred by public funders.^16^ It is possible that funders currently pay more for private and public maternity care than if interventions, particularly caesarean section, were delivered at optimal rates.

To date, there has been little exploration of the actual costs of maternity care to different funders, nor an assessment of the impact that the caesarean section rate may have on these costs. The consideration of costs to different funders is vital when there are a number of funding sources, as is the case in Australia. Identifying total costs or costs to single funders, which has been done in other studies^17-23^ does not identify who actually pays and whether there is any cost-shifting between funders.

 Australia has a mix of public private healthcare. Under the universal healthcare scheme patient treatment within public hospitals for public patients is free-of-charge and funded by the Federal and state governments under public hospital funding agreements. Outside of public hospitals care is partly funded by the Federal government through Medicare with individuals often contributing a co-payment. Prescription pharmaceuticals are also funded by the Federal government through the Pharmaceutical Benefits Scheme (PBS), with patients also paying a small co-payment.

Australia has numerous financial levers to encourage the uptake of private health insurance and the use of private hospital care.^24,25^ These mechanisms are designed to reduce the burden on the public hospital system.^26^ In 2014-2015 within Australia, around half of people aged 18-34 had private health insurance^27^ and in 2015, 27% of women gave birth in a private hospital.^28^ In private hospitals, the hospital *stay* is funded by the private health insurer if the patient holds the appropriate level of coverage. The medical *procedures* performed in the private hospital will be partly funded through Medicare, with private health insurers and/or individuals paying a co-payment. Individuals can also elect to be admitted to a public hospital as a private patient. In this case the *stay* is funded by State and Federal governments, with private insurers also paying a co-payment for the stay; and medical procedures are funded through Medicare, and private health insurers and/or individuals also paying a co-payment. As a result, maternal and early childhood care is provided through a mixture of private and public services, with patients being treated between the 2 systems and a variety of funding sources covering the cost of care.

This paper aims to examine the long-term costs of maternity and early childhood healthcare, over the first 1000 days (defined as covering pregnancy, birth and years post-birth^29^), to different funders of the health system within the state of Queensland, Australia. This paper also aims to identify the role that caesarean section plays in driving costs to different funders. The paper will answer the following research questions:

1. Who funds public and private births and what are the associated costs to different funders of maternal and early childhood healthcare over the first 1000 days?
2. Do the funder and the cost vary by birth type?
3. What are the estimated costs, and to whom, of excess caesarean sections in public and private hospitals?

## Methods

###  Data Source

For this study we utilised a whole of population linked administrative dataset, Maternity1000.^30^ This dataset utilises the Perinatal Data Collection to identify all women who gave birth in the Australian state of Queensland between July 1, 2012 and 30 June, 2015 (n=186789), plus their babies (n=189909). The records of women and babies were then linked to their Admitted Patient Data Collection, Emergency Department Information System, Clinical Costing Unit, and Medicare Benefits Scheme (MBS) and PBS claims records.

###  Study Outcomes

 The primary outcomes for this study were the total cost, and cost to each of the following sources of funding:

1. Public hospitals – paid by Federal and state governments through public hospital funding agreements
2. Medicare – paid by the Federal Government
3. PBS – paid by the Federal Government
4. Private health insurers
5. Individuals

Costs for all health services accessed across the first 1000 days from the estimated date of conception for both mother and child were included. All costs were adjusted to 2017/2018 Australian dollars, based upon Consumer Price Inflation.^31^ Australian dollars are presented throughout. In the Australian healthcare system, women and babies receive care from different providers and in different settings. Because of this, women and babies receive a mix of private and public care, with different funding arrangements.

###  Cost Measurement

The actual cost to public hospital funders of each inpatient event in a public hospital was identified from the Clinical Costing Unit dataset. Costs for emergency department presentations were assigned from the Urgency Related Group code listed for each presentation and the corresponding cost listed on the National Hospital Cost Data Collection (NHCDC) produced by the Independent Hospital Pricing Authority (IHPA).^32^

Costs to private health insurers for the hospital stay of private admissions in public and private hospitals were assigned based on the Australian Refined Diagnosis Related Group code of each admission and the corresponding cost reported by the Private Hospital Data Bureau in their Annual Reports.^33^ Costs to Medicare, and to private health insurers for the medical procedures provided to private patients in public and private hospitals were captured on the MBS claims records.

 Costs to individuals and Medicare for all other services provided outside of hospitals, or as outpatient services, are captured on the MBS claims records. Costs of prescription pharmaceuticals to the PBS and individuals are captured on the PBS claims records.

 The cost of antenatal care was based upon the types of care accessed by each mother and the number of antenatal visits attended, as identified in the Perinatal Data Collection. Women were divided into types of antenatal care and costs assigned as follows:

- Public hospital midwife only: the number of antenatal appointments attended was multiplied by the cost of a midwife antenatal appointment (Tier 2 item 40.28) identified by IHPA on the NHCDC.
- General practitioner (GP) and public hospital midwife only: the number of antenatal appointments was partitioned between GPs and public hospital midwives at a 2:1 ratio, with the cost of GP consultations captured on the MBS dataset, and the cost of midwife consultations taken from the NHCDC (Tier 2 item 40.28).
- Public obstetrician care – no obstetric complications: the number of antenatal appointments was partitioned between public obstetricians and public hospital midwives at a 1:4 ratio, with the cost of public obstetrician consultations taken from the NHCDC for obstetrics consultations for women with no complications (Tier 2 item 20.40), and the cost of midwife consultations taken from the NHCDC.
- Public obstetrician care – with obstetric complications: the number of antenatal appointments was partitioned between public obstetricians and public hospital midwives at a 1:2 ratio, with the cost of public obstetrician consultations taken from the NHCDC for obstetrics consultations for women with complications, and the cost of midwife consultations taken from the NHCDC (Tier 2 item 20.53).
- Private midwife only: it was assumed that the private midwives billed all services through Medicare and thus the costs to Medicare and the patient were captured on the MBS dataset.

###  Public and Private Classification

 All women and babies were classified based upon their intended place of birth, as being either a:

- ‘private birth’ – the baby was born in a private hospital, with mother and baby admitted as private patients. Women and babies who were transferred to a public hospital from a private hospital were classified as a private birth, as this was their intended place of birth;
- ‘public hospital, public birth’ – the baby was born in a public hospital, with mother and baby admitted as public patients;
- ‘public hospital, private birth’ – the baby was born in a public hospital, with mother and baby admitted as private patients.

Planned homebirths were excluded.

###  Analysis of Costs 

 Descriptive statistics are presented to quantify the cost to different funders based on private or public birth type by important time periods in the first 1000 days (antenatal, birth, year 1 for mother, year 1 for baby, year 2 for mother, year 2 for baby) and mode of birth (vaginal birth, instrumental vaginal birth, cesarean section birth).

###  Estimation of the Cost of Excess Caesarean Use

Excess caesarean section use was identified using the World Health Organization’s (WHO’s) C-Model.^34^ The C-Model is a statistical model that calculates a population’s expected caesarean section rate given the population’s characteristics (or ‘case-mix’). Using the published C-Model parameters and each woman’s age, parity, multiple pregnancy, previous caesarean section, gestation at the commencement of labour, fetal presentation, and the onset of labour characteristics ascertained from Maternity1000, each woman’s probability of having a caesarean section was calculated. To identify the number of women who would have had a caesarean section in the counterfactual scenario, women were randomly assigned to have a caesarean section or vaginal birth using Monte Carlo simulation.^35^

 Amongst those who actually had a caesarean section, but were predicted to have a vaginal birth in the counterfactual scenario, the cost of health service use to different funders was estimated. To estimate these costs, the full Maternity1000 dataset was limited to women who had a vaginal birth, and a series of generalised linear models were constructed to estimate the cost to different funders, based upon age, parity, multiple pregnancy, gestation at birth, socioeconomic status, pre-existing medical conditions and if the mother had a private birth. A negative binomial distribution and log link function were specified. Separate models were created for each hospital classification.

In order to produce national estimates, the Maternity1000 dataset was weighted to the Australian population by mother’s age, Indigenous identification, and area of residence. Australian benchmarks for these variables were taken from the Australian Institute of Health and Welfare’s Mothers and Babies reports relating to the years 2012-2015,^28^ to align with each of the years covered by Maternity1000. GREGWT, an algorithm developed by the Australian Bureau of Statistics to weight their population-based surveys, was used to produce the weights.^36^ The mean and summed difference in the actual and counterfactual costs were then presented for public and private births, weighted to the Australian population. All analysis was undertaken in SAS 9.4.

## Results

Between July 1, 2012 and June 30, 2014 in Queensland there 44254 births in a private hospital (28%), 105343 (68%) births in a public hospital with mother and baby admitted as a public patient, and 6113 (4%) births in a public hospital with mother and baby admitted as a private patient. The women had different characteristics based upon the location of birth and funding type. Women who gave birth in private hospitals were older, had a slightly lower body mass index, were less likely to have a medical condition and smoke, and were more likely to be in the highest socioeconomic quintile (Table 1) than women who gave birth in public hospitals. Babies born in private hospitals were less likely to have a 5-minute Apgar score less than 7, and be admitted to the special care nursery or neonatal intensive care unit than babies born in public hospitals. Within public hospitals, women who were admitted as a private patient were slightly older, were less likely to have a medical condition and smoke than women who were admitted as a public patient.

**Table 1:** Socio-Demographic and Infant Characteristics of Women by Birth Location and Funding Type, Queensland, Australia 01/07/2012 – 30/06/2014

**Characteristic**	**Birth Location and Funding Type**		
	**Private Hospital, ** **n = 44254**	**Public Hospital, Public Patient, ** **n = 105343**	**Public Hospital, Private Patient, n = 6113**
	**No. (%)**	**No. (%)**	**No. (%)**
First pregnancy	15196 (34.3)	30030 (28.5)	1809 (29.6)
Singleton pregnancy	42344 (95.7)	102644 (97.4)	5934 (97.1)
Had a medical condition	8877 (20.0)	28088 (26.7)	1269 (20.8)
Smoked before 20 weeks	713 (1.6)	19814 (18.9)	653 (10.8)
Indigenous mother	157 (0.4)	9091 (8.6)	184 (3.0)
SEIFA quintile 1, (lowest)	473 (1.0)	8641 (8.3)	983 (16.2)
SEIFA quintile 2	759 (1.8)	4152 (4.0)	312 (5.1)
SEIFA quintile 3	6304 (14.6)	24485 (23.4)	1306 (21.5)
SEIFA quintile 4	18456 (42.7)	44459 (42.4)	2154 (35.4)
SEIFA quintile 5, (highest)	17219 (39.9)	23021 (22.0)	1333 (21.9)
Had a pregnancy complication	29934 (67.6)	69474 (66.0)	4032 (66.0)
Gestation at birth less than 37 weeks	2363 (5.3)	5924 (5.6)	348 (5.6)
Baby’s 5-minute Apgar score less than 7	643 (1.5)	3096 (2.9)	155 (2.5)
Baby admitted to special care nursery	5786 (13.1)	19465 (18.5)	1124 (18.4)
Baby admitted to neonatal intensive care unit	757 (1.7)	3106 (3.0)	144 (2.4)
Caesarean section birth	21620 (48.9)	29008 (27.5)	2063 (33.8)
Mother’s BMI, mean ± SD	24.6 ± 5.2	25.7 ± 6.3	25.7 ± 5.8
Mother’s age, mean ± SD	32.6 ± 4.5	28.7 ± 5.8	29.9 ± 5.5

Abbreviations: SEIFA, socio-economic index for areas; BMI, body mass index.

The mean cost for women and their babies who gave birth in a private hospital, over the first 1000 days was $24731(Table 2), which was more than the mean cost for those who gave birth in a public hospital as a public patient or a private patient, for whom the mean cost was $22474, and $23494 respectively. Table 2 shows the distribution of these total costs across the different funding sources. On average, the largest contributor to the funding of private hospital births was private health insurers ($11550), followed by Medicare ($7261). For women and their babies who gave birth in public hospitals the largest funding source on average was public hospital funders, contributing $17933 for public patients and $16083 for those who were private patients.

**Table 2:** Mean Costs to Different Funders of Maternity Care by Birth Location and Funding Type Over the First 1000 Days, Queensland, Australia 01/07/2012 – 30/06/2014

**Characteristic**	**Birth Location and Funding Type**		
	**Private Hospital, ** **n = 44254**	**Public Hospital, Public Patient, ** **n = 105343**	**Public Hospital, Private Patient,** **n = 6113**
Total costs	$24731	$22474	$23949
Medicare	$7261	$3539	$4575
PBS	$233	$251	$240
Public hospital funders	$2557	$17933	$16083
Private health insurance	$11550	$272	$1964
Individual out of pocket	$3132	$479	$1087

Abbreviation: PBS, Pharmaceutical Benefits Scheme.

The total costs for women and their babies who gave birth in private hospitals, and for women and their babies who gave birth in public hospitals as public and private patients were highest at the time of birth $10785, $10926 and $11802 respectively; followed by costs in the antenatal time period - $5257, $3352 and $3681 respectively. For all 3 groups (women and their babies who gave birth in private hospitals, and for women and their babies who gave birth in public hospitals as public and private patients) post-birth costs for the mother’s healthcare declined in the first year - $1760, $1666 and $1657 respectively; and then increased in the second year $3131, $2225 and $2458 respectively. Whereas costs for the baby’s health service use was higher in the first year post-birth ($2312, $2749 and $2542) and then declined in the second year postpartum ($1480, $1548 and $1802) (Table 3).

**Table 3:** Mean Costs to Different Funders of Maternity Care by Time of Health Service Use, Queensland, Australia 01/07/2012 – 30/06/2014

**Funding Source**	**Birth Location and Funding Type**		
	**Private Hospital, ** **n = 44254**	**Public Hospital, Public Patient, ** **n = 105343**	**Public Hospital, Private Patient,** **n = 6113**
**Antenatal Care**			
**Total costs**	$5257	$3352	$3681
Medicare	$2506	$1188	$1532
PBS	$36	$36	$32
Public hospital funders	$160	$1900	$1403
Private health Insurance	$590	$38	$165
Individual out of pocket	$1965	$191	$549
**Birth**			
**Total costs**	$10785	$10926	$11802
Medicare	$1552	$133	$588
PBS	$6	$7	$7
Public hospital funders	$576	$10765	$10257
Private health Insurance	$8506	$10	$903
Individual out of pocket	$151	$17	$55
**Mother’s Healthcare – Year 1**			
**Total costs**	$1760	$1666	$1657
Medicare	$760	$582	$630
PBS	$87	$99	$91
Public hospital funders	$183	$921	$617
Private health Insurance	$563	$66	$253
Individual out of pocket	$254	$98	$158
**Baby’s Healthcare – Year 1**			
**Total costs**	$2,312	$2749	$2542
Medicare	$837	$558	$632
Public hospital funders	$837	$2028	$1558
Private health Insurance	$430	$28	$200
Individual out of pocket	$122	$36	$60
**Mother’s Healthcare – Year 2**			
**Total costs**	$3131	$2225	$2458
Medicare	$1061	$649	$747
PBS	$104	$108	$110
Public hospital funders	$257	$1352	$1128
Private health Insurance	$1245	$104	$351
Individual out of pocket	$567	$120	$232
**Baby’s Healthcare – Year 2**			
**Total costs**	$1480	$1548	$1802
Medicare	$544	$428	$446
Public hospital funders	$544	$967	$1120
Private health Insurance	$216	$26	$91
Individual out of pocket	$72	$17	$34

Abbreviation: PBS, Pharmaceutical Benefits Scheme.

There was a large variation in the cost to different funders based upon time period across the first 1000 days. For women and their babies who gave birth in private hospitals, Medicare contributed the largest amount ($2506) in the antenatal time period, followed closely by individuals in out of pocket payments ($1965). Whereas at the time of birth private health insurers contributed the largest amount ($8506). In the first year postpartum Medicare contributed the most to the cost of the mother’s health service use ($760), and Medicare and public hospital funders contributed similar amounts ($837 and $836 respectively) for the baby’s health service use. In the second year post-birth private health insurers contributed the most to the cost of the mother’s health service use ($1245), and Medicare and public hospital funders again contributed similar amounts ($544 and $544 respectively) for the baby’s health service use (Table 3).

For women and their babies who gave birth in public hospitals and were admitted as public patients, costs to public hospital funders were the highest in each time period. For women and their babies who gave birth in public hospitals and were admitted as private patients, costs to public hospital funders was also the highest in each time period, except during the antenatal time period and for the mother’s healthcare costs in the first year post-birth where costs to public hospital funders and Medicare were similar (Table 3).

For women and their babies who gave birth in private hospitals, and in public hospitals as public and private patients the lowest cost mode of birth was vaginal birth without instruments ($20964, $18521 and $18741 respectively); followed by vaginal births with instruments ($22723, $21334 and $22110 respectively). The highest cost mode of birth was caesarean section for all 3 groups ($28244, $31939, and $33257) (Table 4).

**Table 4:** Mean Costs to Different Funders of Maternity Care by Type of Birth Over the First 1000 Days, Queensland, Australia 01/07/2012 – 30/06/2014

**Funding Source**	**Birth Location and Funding Type**		
	**Private Hospital, n = 44254**	**Public Hospital, Public Patient, n = 105343**	**Public Hospital, Private Patient, n = 6113**
**Vaginal Birth, No Instruments**			
**Total costs**	$20,964	$18,521	$18,741
Medicare	$6,210	$3,350	$4,052
PBS	$179	$228	$208
Public hospital funders	$2,144	$14,291	$12,267
Private health insurance	$9,538	$225	$1,299
Individual out of pocket	$2,893	$426	$916
**Vaginal Birth, With Instruments**			
**Total costs**	$22,723	$21,334	$22,110
Medicare	$7,309	$3,738	$4,202
PBS	$150	$196	$132
Public hospital funders	$1,821	$16,483	$15,321
Private health insurance	$10,068	$308	$1,454
Individual out of pocket	$3,375	$609	$1,001
**Caesarean Section Birth**			
**Total costs**	$28,244	$31,939	$33,257
Medicare	$8,091	$3,907	$5,563
PBS	$296	$321	$325
Public hospital funders	$3,068	$26,789	$22,744
Private health insurance	$13,526	$366	$3,226
Individual out of pocket	$3,263	$557	$1,399

Abbreviation: PBS, Pharmaceutical Benefits Scheme.

For women and their babies who gave birth in private hospitals, the costs to Medicare were higher for caesarean section births ($8091) compared to vaginal births with instruments ($7309) and without instruments ($6210). The costs for caesarean sections were also higher for private health insurers ($13526) compared to vaginal births with instruments ($10068) and without instruments ($9538). The costs to public hospital funders were also higher for caesarean sections in private hospitals ($3068) compared to vaginal births with instruments in private hospitals ($1821) and without instruments in private hospitals ($2144) (Table 4).

For women and their babies who gave birth in a public hospital as public patients, the costs to public hospital funders were substantially higher for caesarean sections ($26789) compared to vaginal births with instruments ($16482) and without instruments ($14291). Costs for caesarean sections were also higher for Medicare ($3907) compared to vaginal births with instruments ($3738) and without instruments ($3350) (Table 4).

For women and their babies who gave birth in a public hospital as private patients, the costs to public hospital funders were higher for caesarean sections ($22744) compared to vaginal births with instruments ($15,321) and without instruments ($12267). The costs for caesarean section births were also higher for Medicare ($5563) compared to vaginal births with instruments ($4202) and without instruments ($4052) (Table 4). The costs for caesarean sections were also higher for private health insurers ($3226) compared to vaginal births with instruments ($1454) and without instruments ($1299).

The percentage of women who had a caesarean section birth was higher in private hospitals, with 46% of women who gave birth in private hospitals having a caesarean section birth compared with 27% of women who gave birth in a public hospital as a public patient, and 33% of women who gave birth in a public hospital as a private patient (Table 1). Under the counterfactual scenario, where the probability of having a caesarean section matched the WHO’s C-model across Australia, 30% of women in private hospitals would have a caesarean section, 21% of women who gave birth in public hospitals as a public patient and 24% of women who gave birth in public hospitals as a private patient would have done so. In total, public hospital funders were estimated to have saved $897.7 million for public hospital, public patient births, and $48.0 million for public hospital, private patient births, and $28.1 million for private hospital births. Private health insurers were estimated to have saved $192.2 million and Medicare was estimated to have saved $89.2 million for private hospital births (Table 5).

**Table 5:** Estimated Savings to Different Funders if Caesarean Section Rates Between 2012 and 2014 Were at the Optimal Rate Across Australia, as Estimated by the WHO’s C-Model

**Funding Sources**	**Private Hospital Births, ** **N = 264987**	**Public Hospital, Public Patient Births, ** **N = 601511**	**Public Hospital, Private Patient Births, ** **N = 35334**
Medicare	$89199965	$26733638	$14011992
PBS	$7733636	$6721850	$717212
Public hospital funders	$28129836	$897690963	$48004709
Private health insurance	$192169683	$4965237	$19146,185
Individual out of pocket	$1822186	$6439079	$5838851
Total	$319055306	$942550306	$87718949

Abbreviations: WHO, World Health Organization; PBS, Pharmaceutical Benefits Scheme.

## Discussion

 The average cost of births over the first 1000 days was lowest for public hospital, public patient births ($22474). With private hospital ($24731) and public hospital, private patient ($23949) births costing more on average, reflective of the higher proportion of birth by caesarean section for these women and their babies. For births in private hospitals, private health insurers contributed an average of $11550, which was less than half of the total costs, with Medicare paying $7261 on average and individuals paying $3132 thus making up a large proportion of the total cost. Private health insurers contributed the most at the time of birth ($8506), but only a small amount to the cost of care in pregnancy and in the first and second years after birth. Public hospitals funders were the main contributors to public hospital births, regardless of whether women were admitted as a private or public patient. Caesarean section birth cost all funders more than vaginal births with or without instruments, but particularly public hospital funders, private health insurers and Medicare. However, all funders could stand to make considerable savings – $974 million for public hospital funders, and $216 million for private health insurers – if caesarean section rates across Australia were reduced to more optimal levels.

The results of this study must be considered alongside the limitations of the analyses, the key limitation being the use of the C-Model methodology to quantify the optimal rate of caesarean section use based upon population-level characteristics. It is impossible to accurately identify which caesarean sections were based on medical indications and which were not, without an individual assessment of clinical notes. As such, this paper has relied upon the best available evidence to quantify expected population-level rates. However, it is known that the caesarean section rates in Australia are higher than the average reported for the Organisation for Economic Co-operation and Development,^37^ and that the rates of caesarean section in low risk (selected) age-standardised women in private care in Queensland is almost 1.6 times that of publicly funded women, which is the greatest variation in Australia.^38^ Other studies have also estimated the reduction in public hospital caesarean rates that would be possible if ‘best practice’ was implemented across all hospitals.^39^ An additional weakness of this paper is the use of mean costs to private health insurers per inpatient episode associated with private hospital use, as opposed to identifying the exact amount paid per service. Currently, these data are not available for linkage at the population level. Finally, it should be emphasised that this study sought to describe the costs of current care to different funders identifying how much each funder currently pays, and so unadjusted means are presented. The characteristics of women seeking care under different funding models may explain some of the variation.

This study has demonstrated that caesarean section is the mode of birth that is most costly to all funders. The rates of caesarean section in private and public hospitals are higher than that estimated based upon population-level risk factors, with almost half of women in private care having a caesarean section (47%), despite women with more pregnancy and medical complications giving birth in public hospitals. The higher rate of caesarean section birth in private hospitals has resulted in births, on average costing more in private hospitals than public hospital public patient births. This was similar for the difference in costs between public hospital *public patient* births and public hospital *private patients* births, with rates of caesarean section being higher in the later. However, costs would also have been higher for this group due to the multiple sources of funding public hospitals can claim for private patients. In addition to receiving payment for the activity through public hospital funding agreements, public hospitals and the treating clinicians will also receive funding from private health insurers, Medicare and potentially individuals through out of pocket fees.

The distributional issues associated with public subsidisation of caesarean section in the private sector, with its demonstrated long-term morbidity impacts,^16^ also warrants consideration. While private hospital care may reduce the burden on public hospitals, and thus reduce the cost to public hospitals associated with maternity care, the public still funds a significant portion of private hospital births as this study has demonstrated. Births in private hospitals cost Medicare about twice the amount as public births in public hospitals over the first 1000 days. Furthermore, the overall costs to governments associated with private care are significantly higher than what they would have been had there been lower rates of caesarean section. Recent reforms to Medicare have directly sought to address the overuse of caesarean section in the private sector.^40^ The government rebate to private obstetricians for caesarean section and vaginal birth was equalised in 2017 to remove the previous financial incentive for caesarean sections, and thus reduce costs to Medicare. However, this only covers one occasion of service, and in total the cost to Medicare for caesarean section is considerably higher than other modes of birth.

Recent attention has been given to the overuse of caesarean section worldwide.^41^ Whilst some parts of the world have considerable mortality and morbidity associated with caesarean section not being available when needed, in high-income countries there is the converse: with considerable morbidity experienced by the overuse of the procedure.^16^ It is already known that caesarean section in Australia is associated with additional operating theatre time, specialist obstetrician, anaesthetist, and midwifery care, allied health and pharmacy time and pharmaceutical costs compared to vaginal births.^32^ This paper has highlighted that most of the costs of health service use across the first 1000 days occur at the time of birth, with the cost of caesarean section the largest contributor.

Given that the largest risk factor for having a caesarean section is having had a previous caesarean section,^42^ attention should be given to reducing caesarean section rates in nulliparous women and those with previous caesarean section births. A number of interventions – caseload midwifery,^43^ chart audit^44^ and induction of labour^45^ – have been shown to reduce the risk of caesarean section amongst low-risk nulliparous women. However, caseload midwifery has been shown to be the most cost-effective option.^46^ Caseload midwifery could yield significant cost-savings to all funders. Alternate strategies to reduce the cost of caesarean section, such as reducing the length of stay of patients who have a caesarean section could also be considered.^47^ In addition, the WHO has made a number of recommendations about non-clinical interventions to reduce unnecessary caesarean section, such as antenatal education for women, evidence-based clinical practice guidelines and mandatory second opinion for caesarean section, a collaborative midwifery obstetrician model of care in which the obstetrician provides in-house labour and delivery coverage 24 hours a day, and financial strategies for healthcare professionals or healthcare organisations.^48^

 This study highlights that there is a complex interplay between the costs of maternity care and the outcomes that are produced. Total costs to all funders are lowest on average in public hospital, public patients. The rate of caesarean section deliveries, which are more expensive than other birth types, are higher in private hospitals. All funders, but particularly public hospital, private health insurers and Medicare would see cost savings if caesarean section rates were lower in Australia and in line with optimal rates.

## Ethical issues

 Ethics approval has been obtained from the Townsville Hospital and Health Service Human Research Ethics Committee (HREC) (HREC/16/QTHS/223), and the Australian Institute of Health and Welfare HREC (EO2017-1-338). We have also obtained Public Health Act Approval (RD007377).

## Competing interests

 Authors declare that they have no competing interests.

## Authors’ contributions

 EC conceived and designed the study. EC and HF acquitted the data. EC drafted the manuscript, and undertook the statistical analysis. All authors contributed to the analysis and interpretation of the data and critical revision of the manuscript for important intellectual content.

## Funding

 EC received funding from the National Health and Medical Research Council, NN received funding from the Financial Markets Foundation for Children.

## Authors’ affiliations

^1^Menzies Health Institute Queensland, Griffith University, Southport, QLD, Australia. ^2^Children’s Hospital at Westmead Clinical School, Faculty of Medicine and Health, University of Sydney, Sydney, NSW, Australia. ^3^Department of Maternal Fetal Medicine, Royal Hospital for Women, Randwick, NSW, Australia. ^4^School of Medicine, Griffith University, Southport, QLD, Australia. ^5^School of Nursing and Midwifery, Griffith University, Meadowbrook, QLD, Australia.

## Key Messages

**Implications for policy makers**

- As a result of high caesarean section rates, births in private hospitals are more costly on average than births in public hospitals.
- Although private health insurers pay the most for private hospital births at the time of birth, government funders pay more in the antenatal and postpartum time periods.
- All funders of maternity care would make substantial savings if caesarean section rates were reduced.

**Implications for public**

Private hospital births cost public funders less than public hospital births. However, high rates of caesarean section in private hospitals mean that the public, through public hospital funders, could pay less for private hospital births if caesarean section rates were reduced.
